# Hepatoprotective mechanism of tauroursodeoxycholic acid and its therapeutic potential in liver diseases

**DOI:** 10.3389/fphar.2026.1949645

**Published:** 2026-10-29

**Authors:** Ruting Chao, Yanan Zhang, Yuanzhu Hao, Huihui Zhang, Mingxin Zhang, Chao Sun

**Affiliations:** 1 The First Affiliated Hospital of Xi’an Medical University, Xi’an, China; 2 Graduate School of Xi’an Medical University, Xi’an, China

**Keywords:** bile acids, endoplasmic reticulum stress, liver disease, oxidative stress, TUDCA

## Abstract

Tauroursodeoxycholic acid (TUDCA) is a natural hydrophilic secondary bile acid that acts as an effective chemical chaperone and exerts dramatically hepatoprotective effects. It alleviates endoplasmic reticulum stress (ERS), regulates the unfolded protein response (UPR), suppresses inflammatory responses, reduces oxidative stress, and inhibits hepatocyte apoptosis. In addition, TUDCA maintains bile acid metabolism homeostasis and modulates the gut–liver axis, showing significant therapeutic potential in multiple liver disorders, including metabolic dysfunction-associated steatotic liver disease (MASLD), cholestatic liver injury, liver fibrosis, hepatocellular carcinoma (HCC), hepatic ischemia–reperfusion injury (IRI). Despite promising preclinical evidence, the specific crosstalk between TUDCA and the gut microbiota remains largely unclear, and high-quality clinical evidence supporting its translation into clinical practice is still limited. This narrative review summarises the molecular targets and signaling pathways of TUDCA, discusses current research gaps, and provides a theoretical foundation for its rational clinical application and future mechanistic studies.

## 1 Introduction

As the largest solid organ of the human body, the liver performs a central function in the metabolism, synthesis and conversion of nutrients, alongside bile production, detoxification and bodily defense. In recent years, the incidence of liver diseases such as MASLD, cholestatic liver disease, and HCC has gradually risen, becoming a major public health challenge in China and globally in addition to imposing a heavy burden on healthcare resources.

Synthesized by hepatocytes from cholesterol, bile acids are the primary constituents of bile (Fuchs et al., 2025). Hepatocytes use cholesterol as a precursor to synthesise primary bile acids; once secreted into the intestine, the gut microbiota convert these primary bile acids into secondary bile acids (Fuchs and Trauner, 2022). TUDCA is the taurine-conjugated form of ursodeoxycholic acid UDCA, a hydrophilic secondary bile acid (Cabrera et al., 2019). TUDCA has the molecular formula C_26_H_45_NO_6_S and a molecular weight of approximately 499.7 g/mol (Huang et al., 2024). The chemical structures of UDCA and its taurine-conjugated derivative TUDCA are shown in Figure 1.

**FIGURE 1:**
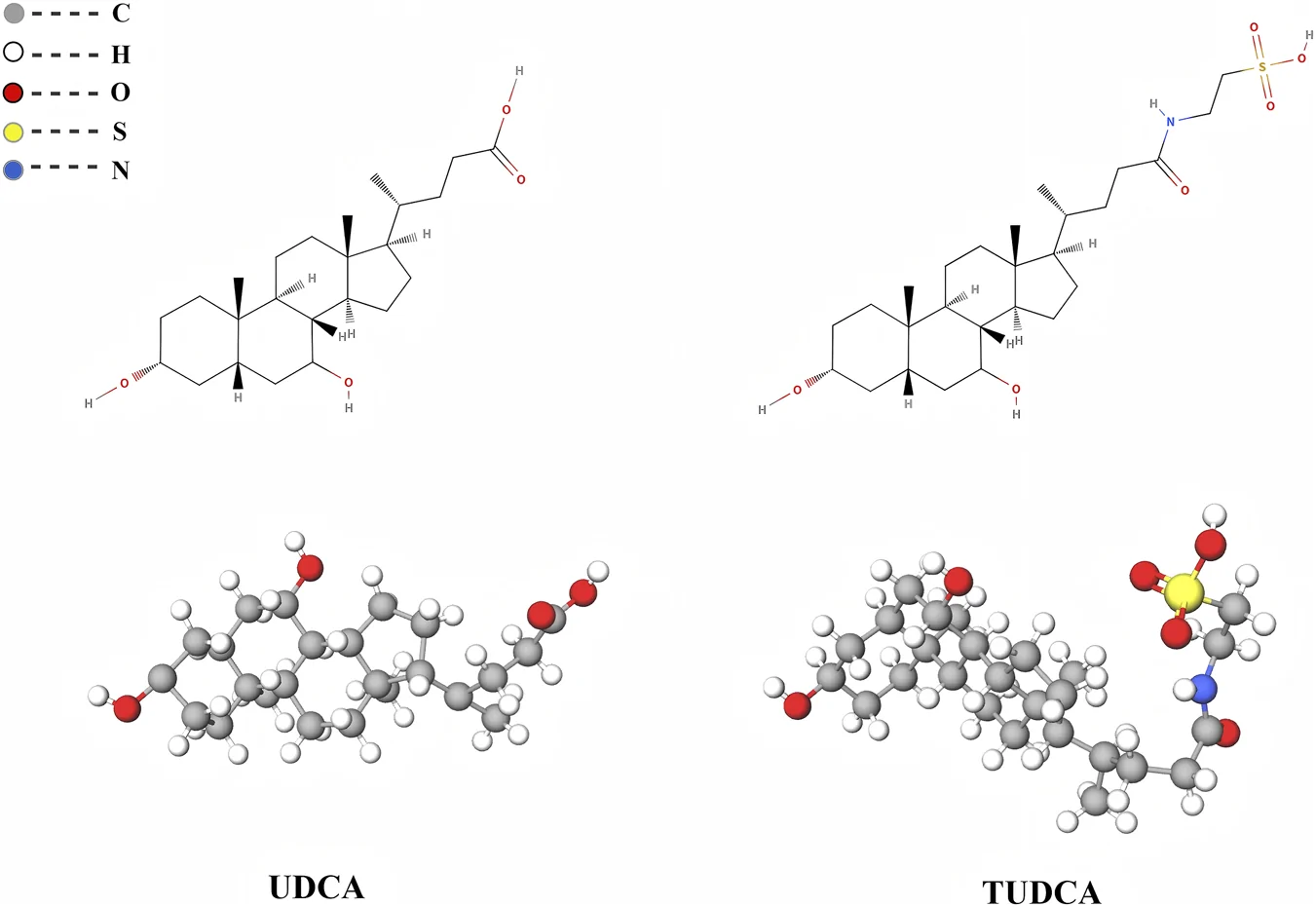
Chemical structures of UDCA and TUDCA. Two-dimensional and three-dimensional representations of UDCA and its taurine-conjugated derivative TUDCA are shown. Abbreviations: TUDCA, tauroursodeoxycholic acid; UDCA, ursodeoxycholic acid.

UDCA and TUDCA naturally exist in the bile acids of hibernating bears. Both have liver-protective, detoxifying, and gallstone-dissolving effects and a long history of use in traditional Chinese medicine (Herta and Beuers, 2022). Research has shown that UDCA has a hepatoprotective effect and can promote the production and flow of bile. Approved by the US Food and Drug Administration (FDA), UDCA is indicated for primary biliary cholangitis (PBC) (Vang et al., 2014). As the taurine-conjugated form of UDCA, TUDCA has attracted growing attention for its cytoprotective properties. Experimental studies have shown that TUDCA can modulate ERS, apoptosis, inflammatory responses, and other cellular processes associated with liver injury. These findings have generated increasing interest in the potential hepatoprotective effects of TUDCA, although their clinical relevance remains to be fully established.

Previous reviews have summarized the biological properties, mechanisms of action, and therapeutic applications of hydrophilic bile acids, including UDCA and TUDCA. In contrast, others have focused primarily on the general molecular and cytoprotective effects of TUDCA. However, TUDCA has often been discussed together with other bile acid derivatives or outside a disease-specific hepatic context. More recent studies have expanded the evidence regarding TUDCA across metabolic, cholestatic, fibrotic, ischemic, and malignant liver disease models. An updated liver-focused review that relates these mechanistic findings to disease-specific experimental and clinical evidence is therefore warranted.

Accordingly, this review summarizes current knowledge regarding the hepatoprotective mechanisms of TUDCA and discusses its potential therapeutic implications across major liver diseases. Particular attention is given to the context and strength of the available evidence, as well as to unresolved questions that currently limit clinical translation.

## 2 Literature search strategy

Literature relevant to this review was identified through PubMed, Web of Science, and the China National Knowledge Infrastructure (CNKI). The search focused primarily on studies published during the 15 years up to 2026, with earlier studies included when they provided important mechanistic, clinical, or pharmacokinetic evidence. Search terms centered on “tauroursodeoxycholic acid” and “TUDCA,” combined with terms related to liver disease and specific conditions such as metabolic dysfunction-associated steatotic liver disease, cholestatic liver disease, and hepatocellular carcinoma, together with earlier disease terminology and corresponding Chinese terms where appropriate. Original studies directly examining TUDCA in hepatic or hepatobiliary conditions were prioritized, while extrahepatic studies were included when they provided relevant mechanistic evidence.

## 3 The core molecular mechanism of TUDCA interaction with cells

### 3.1 The biosynthesis of TUDCA

The biosynthesis of TUDCA commences with cholesterol as the starting material and proceeds through a series of enzymatic reactions, in conjunction with gut microbiota metabolism. The primary pathway involves cholesterol transformed into 7α-hydroxycholesterol mediated by microsomal cholesterol 7α-hydroxylase (CYP7A1), which is further catalyzed by 3β-hydroxysteroid dehydrogenase to yield 7α-hydroxy-4-cholesten-3-one. A series of catalytic reactions then takes place, with mitochondrial sterol 27-hydroxylase catalysing cleavage of the steroid side chain by making the final output CDCA. In addition, there is a bypass metabolic pathway: Cholesterol initially undergoes enzymatic modification by sterol 27-hydroxylase (CYP27A1) to produce 27-hydroxycholesterol. Subsequent hydroxylation driven by sterol 7α-hydroxylase creates 3β-7α-dihydroxy-5-cholestenoic acid, and the last catalytic reaction carried out by CYP27A1 ultimately gives rise to CDCA (4).

CDCA, along with its conjugated derivatives, is excreted into the colon with bile, where intestinal microbes metabolize it to generate UDCA. This microbial conversion is mediated by a C7-hydroxyl epimerization process, in which bacterial 7α-hydroxysteroid dehydrogenase 7α‐HSDH first oxidizes CDCA to form 7-ketolithocholic acid (7-KLCA), followed by reduction of this intermediate by 7β-hydroxysteroid dehydrogenase 7β‐HSDH to produce UDCA. The newly formed UDCA is reabsorbed into the liver via enterohepatic circulation and subsequently undergoes taurine conjugation in hepatocytes to form TUDCA (Vavrusáková et al., 2025; Song et al., 2023). The overall biosynthetic pathway of TUDCA, from cholesterol to the final taurine-conjugated product, is summarized in Figure 2.

**FIGURE 2:**
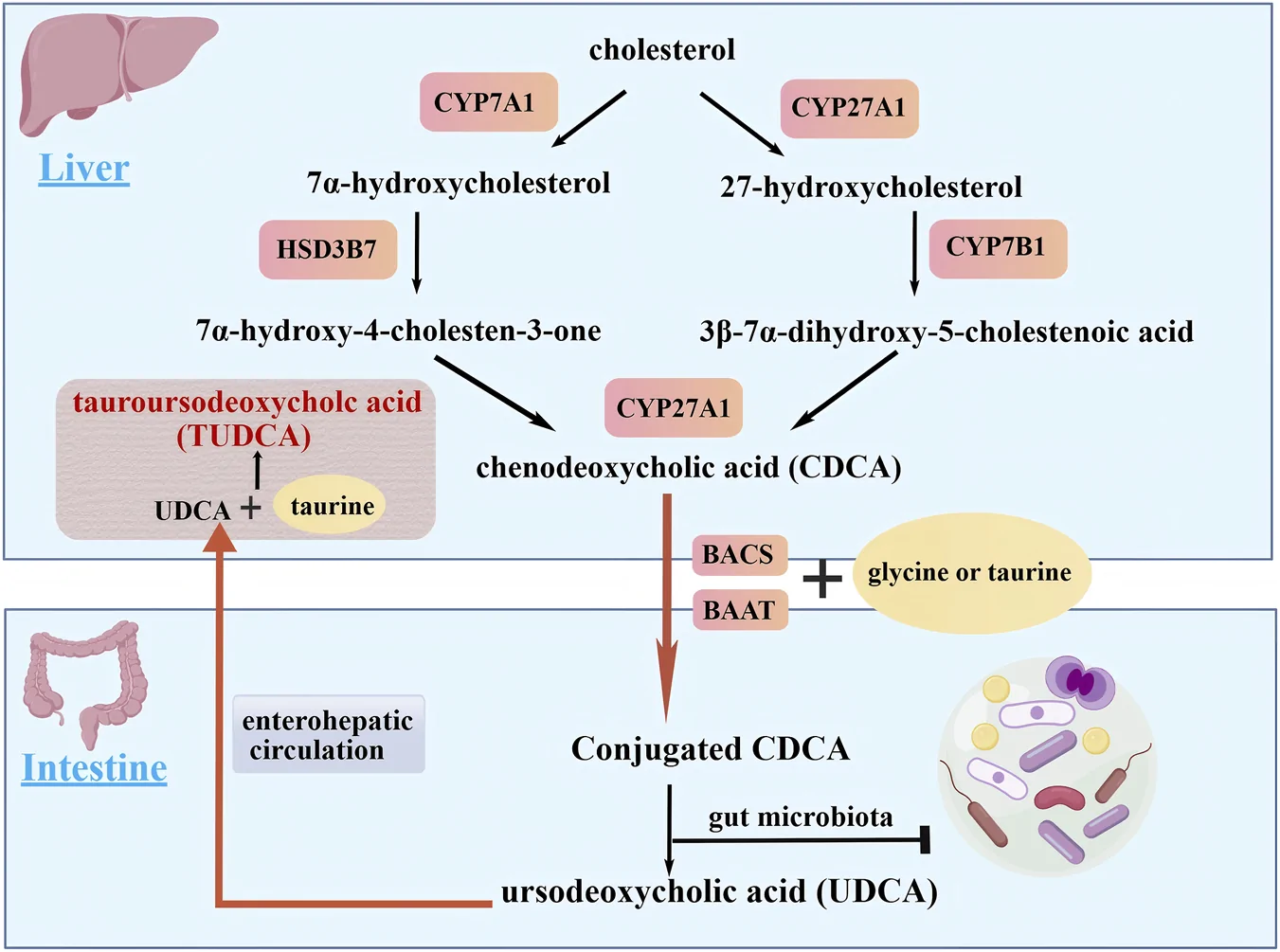
Biosynthesis and enterohepatic formation of TUDCA. Cholesterol is converted to CDCA through the classical and alternative bile acid synthesis pathways in the liver. Following conjugation and intestinal microbial transformation, UDCA is formed and returned to the liver through enterohepatic circulation, where conjugation with taurine generates TUDCA. Abbreviations: BAAT, bile acid-CoA:amino acid N-acyltransferase; BACS, bile acid-CoA synthetase; CDCA, chenodeoxycholic acid; CYP7A1, cholesterol 7α-hydroxylase; CYP7B1, oxysterol 7α-hydroxylase; CYP27A1, sterol 27-hydroxylase; HSD3B7, 3β-hydroxysteroid dehydrogenase type 7; TUDCA, tauroursodeoxycholic acid; UDCA, ursodeoxycholic acid.

### 3.2 Regulating endoplasmic reticulum stress and the unfolded protein response

The endoplasmic reticulum (ER) constitutes a central component of the endomembrane system. It mediates multiple vital biochemical processes, including protein folding, post-translational modification, and lipid biosynthesis (Xia et al., 2020; Ajoolabady et al., 2022). Accordingly, maintaining its homeostasis is an essential prerequisite for normal physiological function in cells and tissues (Chipurupalli et al., 2019). However, when cells are exposed to stress stimuli like nutrient starvation, oxidative stress, or viral infection (Alotaibi and Alkhammash, 2025), the ER’s ability to fold proteins is compromised, and protein homeostasis is disrupted. Consequently, an abnormal buildup of misfolded and unfolded proteins accumulates within the ER lumen, a pathological condition known as ERS (Chen et al., 2023).

The UPR is a complex adaptive signaling pathway modulated by three transmembrane sensors, including the PKR-like endoplasmic reticulum kinase (PERK), inositol-requiring enzyme 1α (IRE1α), and activating transcription factor 6 (ATF6) (Jeon and Ronai, 2026). Under resting conditions, these transmembrane receptors stay inactive because they interact with Glucose-Regulated Protein 78 (GRP78), a molecular chaperone. However, once ER homeostasis is compromised, GRP78 dissociates from these three transmembrane receptors, thereby leading to their activation (Sadeghipour et al., 2023). This relieves the stress through the modulation of cellular processes, such as protein synthesis reduction, acceleration of protein degradation, and promotion of chaperone expression, leading to protein homeostasis (Spencer and Finnie, 2020).

The liver is a vital biosynthetic organ and is easily affected by ERS (Steinberger et al., 2022). Recent studies have confirmed that ERS plays a pivotal role in driving various hepatic pathologies, including MASLD, IRI, and HCC (Latif et al., 2022; Obert et al., 2019; Vandewynckel et al., 2015). TUDCA is widely used experimentally as a chemical chaperone (Kusaczuk, 2019). In a chronic intermittent hypoxia-induced liver injury model, TUDCA significantly downregulates GRP78 expression and reduces PERK, IRE1α, and ATF6, thereby inhibiting ERS activation at its source. Furthermore, TUDCA effectively reverses the abnormal overexpression of ERS-induced apoptosis-promoting proteins, including C/EBP homologous protein (CHOP), activated caspase-12, phosphorylated Eukaryotic Initiation Factor 2α, and phosphorylated JNK induced by chronic intermittent hypoxia (CIH), thereby blocking the transmission of ERS signals to apoptosis and exerting its hepatoprotective effects (Hou et al., 2017).

### 3.3 Anti-inflammation

The inflammatory response plays a pivotal role in various hepatic disorders, including MASLD, hepatic IRI, HCC, and CIH-induced liver injury (Obert et al., 2019; Hou et al., 2017; Ye et al., 2021; Lebeaupin et al., 2015).

To date, multiple studies have confirmed that ERS is intrinsically linked to the onset of inflammatory responses, influencing their development through JNK-AP-1-, NF-κB-, and IκB kinase (IKK)-dependent pathways (Kusaczuk, 2019). Research indicates that ERS induces activation of the hepatic NLRP3 inflammasome, subsequently giving rise to hepatocyte death. TUDCA can prevent this cell death by inhibiting ER-dependent inflammasome activation (Lebeaupin et al., 2015; Baba et al., 2024). A tunicamycin-induced model in male rats resulted in marked hepatotoxicity and liver dysfunction. Exogenous administration of TUDCA significantly downregulated cytoplasmic phospholipase A2 and cyclooxygenase-2 expression in hepatic tissue and inhibited prostaglandin E2 production, ultimately leading to marked recovery of serum alanine aminotransferase (ALT), aspartate aminotransferase (AST), and total bilirubin (TBIL) levels (Aslan et al., 2021; Aslan et al., 2018). In a mouse model of NASH, as defined in the original study, the long non-coding RNA gm9795 induces ERS, activates the NF-κB and JNK pathways, and promotes inflammatory cascades; TUDCA is able to effectively dampen this process. Concurrently, TUDCA markedly downregulates the generation of pro-inflammatory cytokines, notably TNF-α, IL-6, and IL-1β (Ye et al., 2021; Wang et al., 2018).

### 3.4 Antioxidant

TUDCA also exerts cytoprotective effects through its antioxidant properties. As the central hub of metabolism in eukaryotic cells, the functional homeostasis of mitochondria is highly susceptible to disruption by ERS (Teodoro et al., 2022). ERS can further impair mitochondrial function by inducing mitochondrial calcium dysregulation, leading to excessive reactive oxygen species (ROS) production and oxidative damage. In hepatic injury models, TUDCA has been shown to alleviate oxidative stress through multiple mechanisms. In a mouse model of fatty liver ischemia–reperfusion injury, TUDCA reduced ERS-induced mitochondrial calcium overload in hepatic macrophages, thereby decreasing mitochondrial ROS production and suppressing ROS-dependent NLRP3 inflammasome activation, ultimately attenuating inflammatory liver injury (Li et al., 2024). Furthermore, studies in obese mice and hepatocyte models demonstrated that TUDCA enhances endogenous antioxidant defenses by inhibiting the interaction between KEAP1 and NRF2, promoting NRF2 nuclear translocation, and increasing the expression of downstream antioxidant genes, including Nqo1, Ho-1, and Gpx4, thereby reducing intracellular ROS accumulation (Zhang et al., 2022).

Beyond hepatic models, extrahepatic studies have provided additional insights into the conserved antioxidant properties of TUDCA. For example, in retinal neural cells exposed to high-glucose stress, TUDCA reduced ROS production and oxidative protein damage, suggesting a broader cytoprotective mechanism involving regulation of oxidative stress (Gaspar et al., 2013). However, findings from extrahepatic systems should be interpreted as mechanistic support for TUDCA’s antioxidant capacity rather than as direct evidence of hepatoprotection.

### 3.5 Anti-apoptosis

TUDCA exerts anti-apoptotic effects in hepatic injury by regulating mitochondrial-dependent cell death and pro-survival signaling pathways. In primary rat hepatocytes exposed to glycochenodeoxycholic acid (GCDCA), TUDCA reduced caspase-9 and caspase-3 activation as well as apoptotic nuclear changes. Mechanistically, this protective effect was associated with activation of the p38 MAPK, ERK1/2 MAPK, and PI3K signaling pathways (Schoemaker et al., 2004). In the same hepatocyte model, inhibition of NF-κB did not diminish the protective effect of TUDCA, suggesting that its anti-apoptotic action under GCDCA-induced stress is largely independent of NF-κB activation (Schoemaker et al., 2004). This differs from the inhibitory effect of TUDCA on NF-κB signaling observed in inflammatory liver models, suggesting that the role of NF-κB may vary with cellular context and injury type.

Beyond hepatic models, extrahepatic studies have provided additional mechanistic insights into the anti-apoptotic actions of TUDCA. In amyloid-β-treated PC12 neuronal cells, TUDCA attenuated E2F-1 induction, p53 stabilization, and Bax expression, thereby suppressing the E2F-1/p53/Bax apoptotic pathway (Ramalho et al., 2004). In primary cortical neurons, TUDCA-mediated protection was associated with PI3K/Akt-dependent survival signaling, reduced Bax translocation to mitochondria, decreased cytochrome c release, and subsequent inhibition of caspase activation (Solá et al., 2003). Nuclear steroid receptor-dependent regulation has also been reported in neuronal models, in which TUDCA promoted dissociation of the mineralocorticoid receptor from HSP90 and its subsequent nuclear translocation (Solá et al., 2006). These findings suggest that TUDCA may regulate apoptosis through multiple complementary pathways, although the contribution of some of these mechanisms to hepatoprotection remains to be further clarified.

## 4 Research into the application of TUDCA in common liver diseases

### 4.1 Metabolic dysfunction-associated steatotic liver disease

In recent years, the global prevalence of MASLD has risen rapidly due to changes in lifestyle. This condition has become the world’s most common chronic hepatic disease, affecting nearly one-quarter of adults across the globe (Wade et al., 2024; Yang et al., 2025). Relevant investigations have corroborated that the progression of MASLD is in close relation with lipid accumulation, oxidative stress and ERS (Heeren and Scheja, 2021; Guo et al., 2022).

The effects of TUDCA on ERS have been explored at both the cellular and animal levels. In AML12 hepatocytes, TUDCA attenuated lncRNA-Gm9795-associated ERS and reduced activation of the IRE1α/NF-κB/JNK pathway (Ye et al., 2021). Similar effects were observed in Lis1-deficient primary hepatocytes, in which TUDCA reduced ERS markers and intracellular lipid accumulation, further linking ERS relief to improved hepatocellular lipid homeostasis (Li et al., 2018a). In addition, NFATc1 has been identified as an important mediator of chronic ERS in fatty liver disease. In primary hepatocytes and AML12 cells, TUDCA suppressed NFATc1-associated terminal UPR signaling, including the PERK/PKR–eIF2α–CHOP pathway and downstream NLRP3 inflammasome activation (Latif et al., 2022). These findings were further supported in Western diet-fed mice, in which TUDCA treatment was associated with reduced terminal UPR signaling, hepatic inflammation, apoptosis, and fibrosis (Latif et al., 2022).

TUDCA has also shown lipid-lowering and antisteatotic effects in animal models. In HFD-induced hyperlipidemic mice, TUDCA improved circulating lipid profiles and attenuated hepatic lipid deposition and steatotic changes (Cui et al., 2023). In another mouse model of MASLD induced by a high-fat diet, TUDCA reduced long-chain fatty acid uptake by hepatocytes by inhibiting the expression of the farnesoid X receptor (FXR) and fatty acid transporter 5 (FATP5), thereby alleviating lipid accumulation (Wang et al., 2024). In parallel, in an HFD-induced NAFLD model, as defined in the original study, TUDCA partially restored gut microbial composition toward that observed in control mice. TUDCA increased the relative abundance of potentially anti-inflammatory taxa, including *Faecalibacterium* and *Akkermansia*, while reducing *Mucispirillum and Ruminococcus gnavus*. These microbiota changes were accompanied by attenuation of intestinal inflammation, improvement of intestinal barrier function, and reduced hepatic steatosis (Wang et al., 2018).

Preliminary clinical findings are broadly consistent with these experimental observations. In a 24-week study of 186 patients with NAFLD under the previous nomenclature, TUDCA combined with polyene phosphatidylcholine produced greater reductions in liver enzymes and controlled attenuation parameter (CAP) than polyene phosphatidylcholine combined with glutathione (Lu et al., 2022). An earlier randomized study of 180 patients also reported improvements in liver biochemical indices, lipid profiles, and ultrasonographic findings with a TUDCA-containing regimen (Cao et al., 2013). These studies provide initial clinical support for the potential benefit of TUDCA in fatty liver disease, although the use of combination therapy and predominantly surrogate endpoints limits conclusions regarding its independent and long-term efficacy in MASLD.

The effects of TUDCA on FXR also appear to depend on the pathophysiological context. While reduced hepatic FXR expression was associated with decreased FATP5 expression and fatty acid uptake in HFD-induced fatty liver (Wang et al., 2024), TUDCA increased nuclear FXR and NRF2 levels in experimental cholestasis and promoted bile acid export through regulation of BSEP, MRP2, NTCP, and CYP7A1 (Song et al., 2023). Notably, TUDCA retained hepatoprotective effects in Fxr-deficient mice through NRF2-associated signaling (Zhang et al., 2023), suggesting that TUDCA does not simply act as a uniform FXR agonist or antagonist, and that its influence on FXR-related pathways may vary with the metabolic and bile acid environment. The proposed mechanisms are summarized in Figures 3, 4.

**FIGURE 3:**
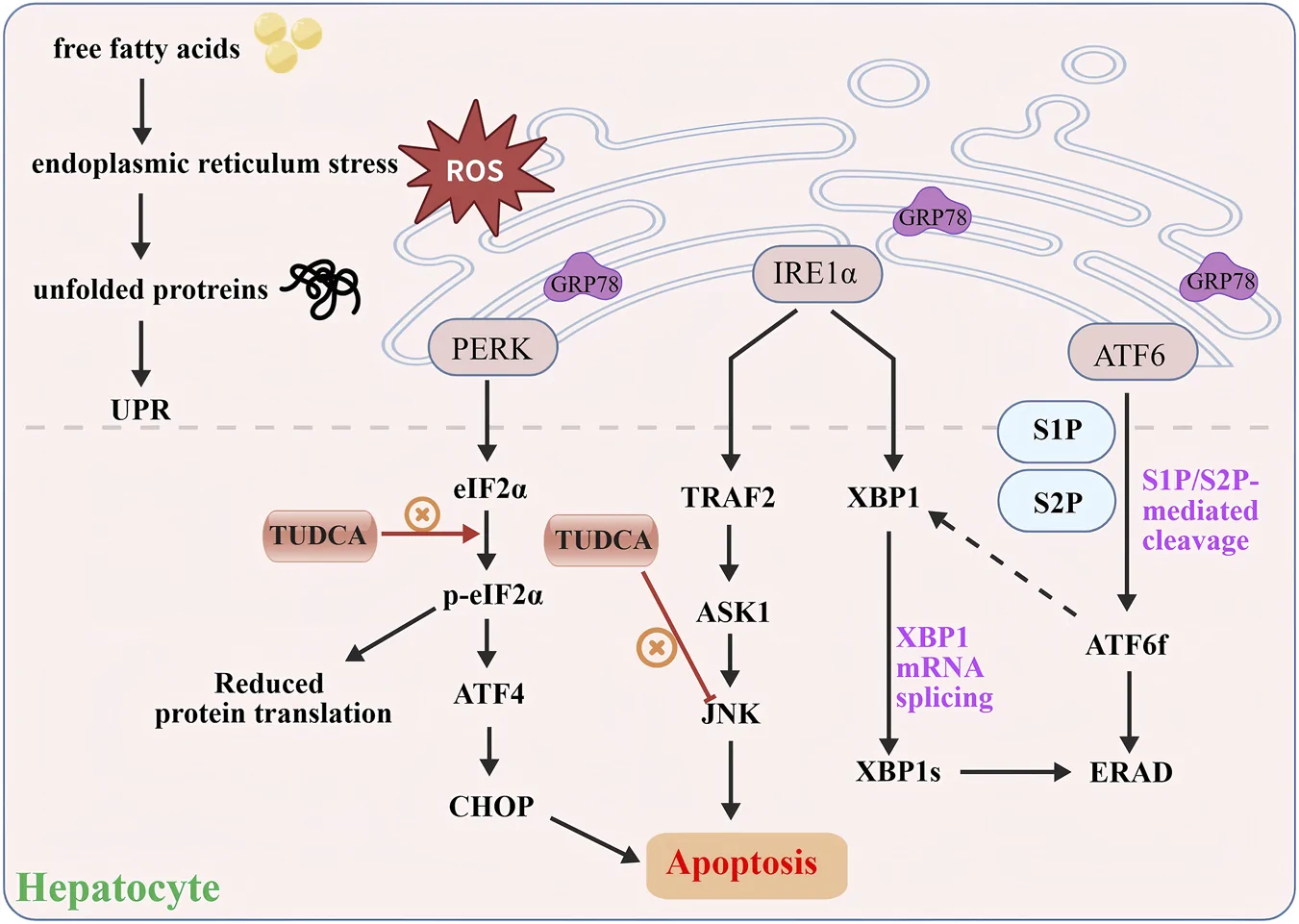
Modulation of ERS and UPR signaling by TUDCA in MASLD. Free fatty acid-induced ERS promotes the accumulation of unfolded proteins and activates the PERK, IRE1α, and ATF6 branches of the UPR. TUDCA attenuates ERS-related signaling, including the PERK/eIF2α/CHOP and IRE1α/TRAF2/ASK1/JNK pathways, thereby limiting pro-apoptotic responses. Abbreviations: ASK1, apoptosis signal-regulating kinase 1; ATF4, activating transcription factor 4; ATF6, activating transcription factor 6; ATF6f, cleaved active form of ATF6; CHOP, C/EBP homologous protein; eIF2α, eukaryotic translation initiation factor 2α; ER, endoplasmic reticulum; ERAD, ER-associated degradation; GRP78, glucose-regulated protein 78; IRE1α, inositol-requiring enzyme 1α; JNK, c-Jun N-terminal kinase; MASLD, metabolic dysfunction-associated steatotic liver disease; PERK, protein kinase RNA-like ER kinase; p-eIF2α, phosphorylated eIF2α; ROS, reactive oxygen species; S1P, site-1 protease; S2P, site-2 protease; TRAF2, TNF receptor-associated factor 2; TUDCA, tauroursodeoxycholic acid; UPR, unfolded protein response; XBP1, X-box binding protein 1; XBP1s, spliced X-box binding protein 1.

**FIGURE 4:**
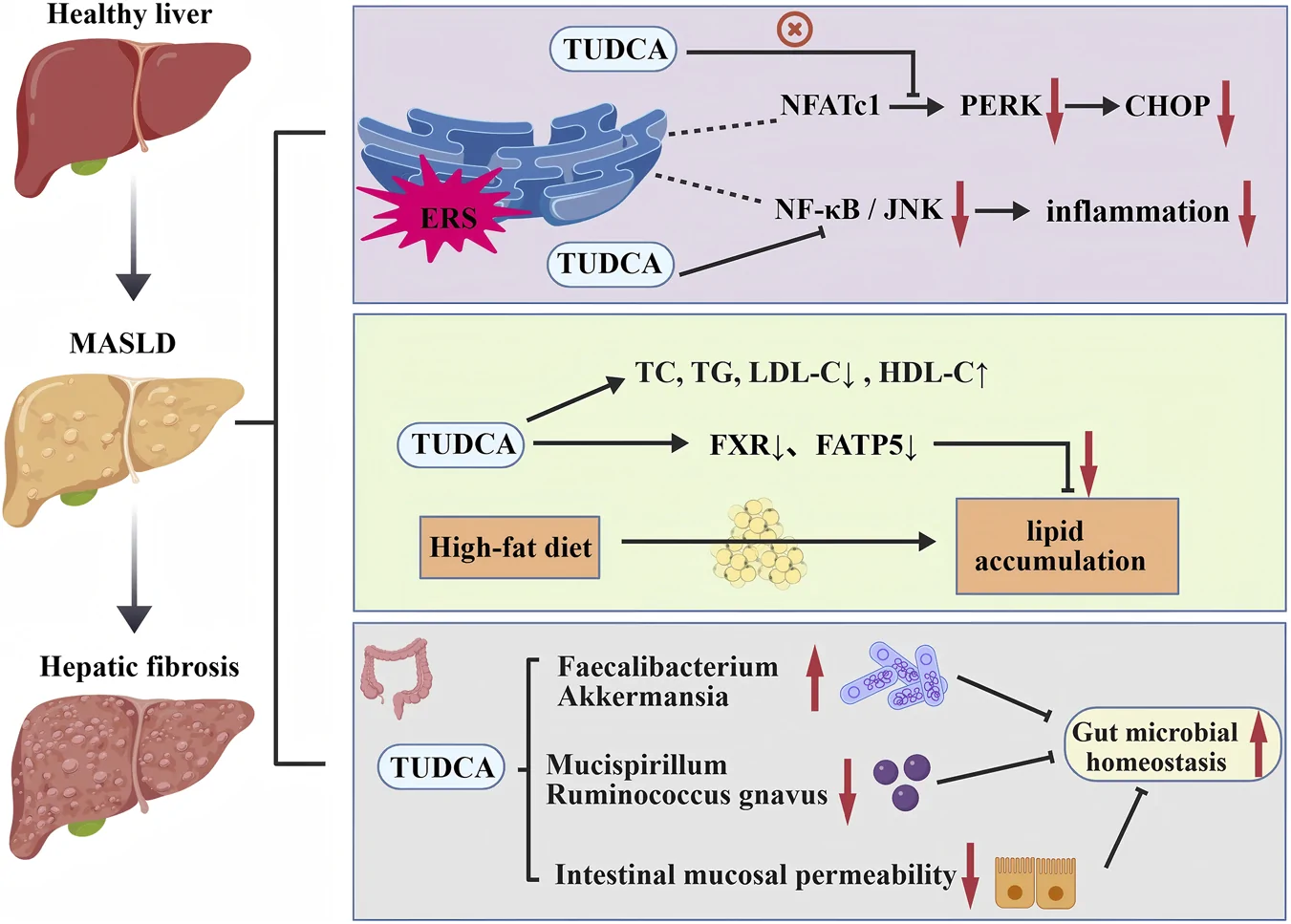
Proposed protective mechanisms of TUDCA in MASLD. TUDCA may attenuate MASLD-associated injury by modulating ERS-related PERK/CHOP and NF-κB/JNK signaling, reducing hepatic lipid accumulation through FXR/FATP5-associated pathways, and altering gut microbial composition and intestinal mucosal permeability. Abbreviations: CHOP, C/EBP homologous protein; ERS, endoplasmic reticulum stress; FATP5, fatty acid transport protein 5; FXR, farnesoid X receptor; HDL-C, high-density lipoprotein cholesterol; JNK, c-Jun N-terminal kinase; LDL-C, low-density lipoprotein cholesterol; MASLD, metabolic dysfunction-associated steatotic liver disease; NFATc1, nuclear factor of activated T cells 1; NF-κB, nuclear factor kappa B; PERK, protein kinase RNA-like ER kinase; TC, total cholesterol; TG, triglycerides; TUDCA, tauroursodeoxycholic acid.

### 4.2 Cholestatic liver injury

Cholestasis represents a pathophysiological state induced by dysfunction in bile secretion and elimination,and encompasses a wide range of diseases of the hepatobiliary system. PBC represents a chronic autoimmune hepatic disorder that mainly targets small intrahepatic bile ducts, manifested as biliary stasis and hepatic fibrosis, which may ultimately progress to cirrhosis and/or liver failure (Chen et al., 2026). At the cellular level, increased expression of the ERS markers PDI and GRP78 has been observed in damaged small bile ducts from patients with PBC, while TUDCA pretreatment suppressed ERS, deregulated autophagy, and cellular senescence in cultured biliary epithelial cells (Sasaki et al., 2015). These findings suggest that modulation of ERS may contribute to the protective effects of TUDCA in PBC.

Clinical evidence in PBC further supports its therapeutic potential. Currently, UDCA is one of the therapeutic regimens recommended in guidelines for PBC patients. TUDCA, as the taurine-conjugated form of UDCA, exhibits enhanced water solubility and is more readily absorbed and utilized by the intestine and liver (Pan et al., 2013). In a multicenter, randomized, double-blind trial involving 199 patients with PBC, both TUDCA and UDCA were administered at 250 mg three times daily for 24 weeks. A reduction in serum alkaline phosphatase (ALP) of more than 25% was achieved in 75.97% of patients receiving TUDCA and 80.88% receiving UDCA, with similar changes in ALP, AST, and total bilirubin and comparable safety between the two groups (Ma et al., 2016). However, the available comparative clinical evidence remains limited, and this trial was relatively short and relied predominantly on biochemical endpoints. Therefore, the current data support comparable short-term biochemical efficacy and safety of TUDCA and UDCA rather than demonstrating superiority of TUDCA or an established advantage in long-term clinical outcomes.

Experimental cholestasis models have provided further mechanistic evidence. In mice with cholic acid (CA)- or α-naphthyl isothiocyanate (ANIT)-induced cholestasis, TUDCA improved bile acid homeostasis through hepatic FXR and NRF2 signaling. In the CA model, TUDCA increased nuclear FXR and NRF2 levels, enhanced BSEP and MRP2 expression, and reduced NTCP and CYP7A1 expression (Song et al., 2023). Its protective effect was also retained in Fxr-deficient mice through NRF2-dependent regulation, indicating that TUDCA is not exclusively dependent on FXR signaling. In both CA- and ANIT-induced cholestasis, TUDCA reduced GRP78 and CHOP expression and inhibited the CHOP–DR5–caspase-8 apoptotic pathway (Zhang et al., 2023). Beyond experimental cholestasis, TUDCA has also shown protective effects in secondary biliary fibrosis. In a common bile duct ligation model, TUDCA reduced fibrotic and apoptotic injury through modulation of hepatic UPR signaling (Paridaens et al., 2017), providing complementary evidence that is discussed further in the liver fibrosis section. A schematic overview of the protective mechanisms of TUDCA in cholestatic liver injury is presented in Figure 5.

**FIGURE 5:**
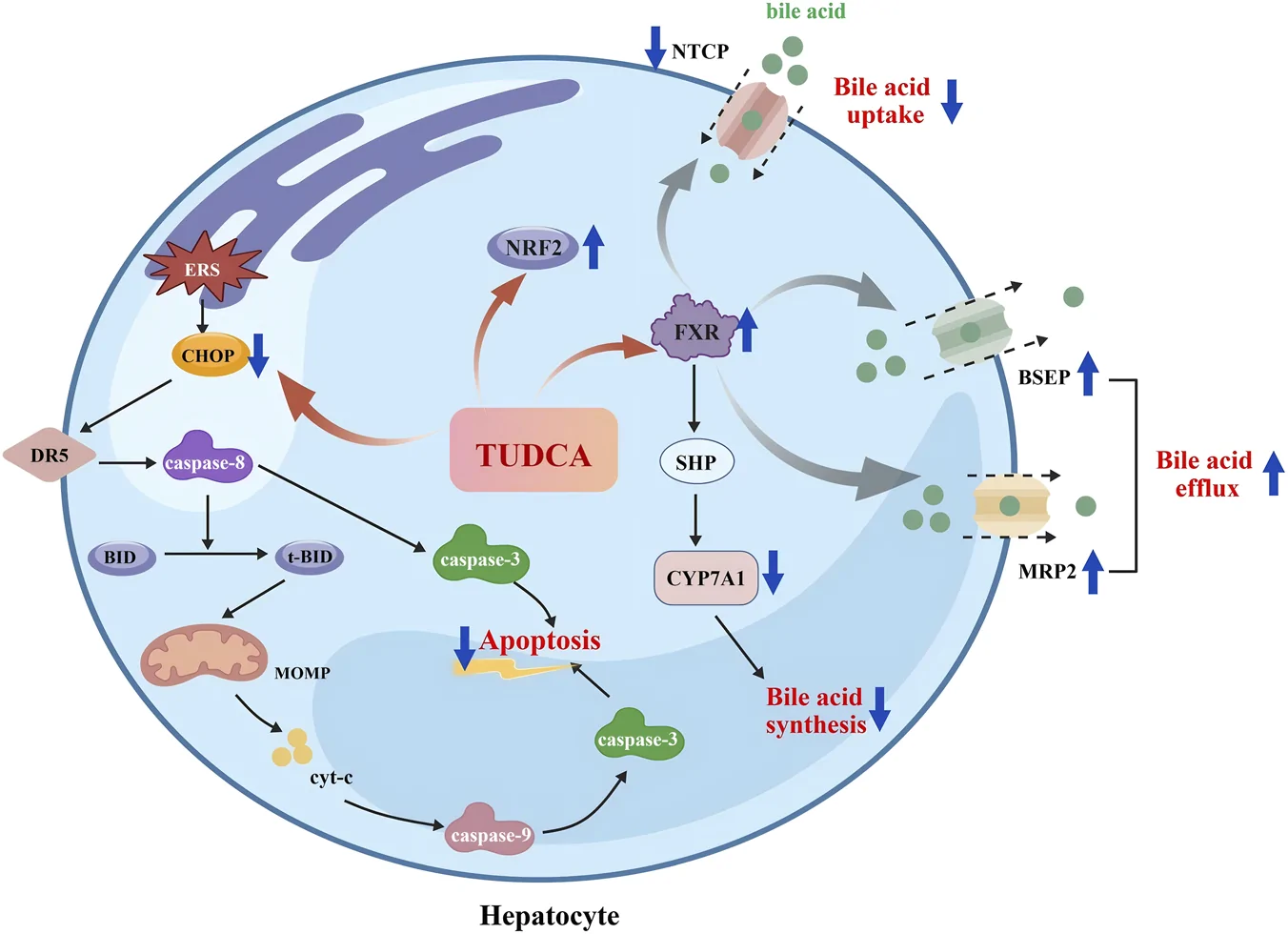
Proposed protective mechanisms of TUDCA in cholestatic liver injury. TUDCA modulates FXR- and NRF2-related signaling and bile acid homeostasis, with reduced NTCP and CYP7A1 expression and increased BSEP and MRP2 expression, thereby limiting bile acid uptake and synthesis while promoting bile acid efflux. NRF2 may also contribute to BSEP and MRP2 regulation independently of FXR. TUDCA further attenuates ERS-associated apoptosis through suppression of the CHOP–DR5–caspase-8 pathway. Abbreviations: BID, BH3-interacting domain death agonist; BSEP, bile salt export pump; CHOP, C/EBP homologous protein; CYP7A1, cholesterol 7α-hydroxylase; cyt-c, cytochrome c; DR5, death receptor 5; ERS, endoplasmic reticulum stress; FXR, farnesoid X receptor; MOMP, mitochondrial outer membrane permeabilization; MRP2, multidrug resistance-associated protein 2; NRF2, nuclear factor erythroid 2-related factor 2; NTCP, sodium taurocholate cotransporting polypeptide; SHP, small heterodimer partner; t-BID, truncated BID; TUDCA, tauroursodeoxycholic acid.

### 4.3 Liver fibrosis

Liver fibrosis is characterized by excessive extracellular matrix deposition and is a common consequence of chronic liver injury that may progress to cirrhosis and HCC (Schuppan et al., 2018). Preclinical studies suggest that TUDCA can attenuate fibrotic injury by modulating ERS, apoptosis, and liver regeneration. In a CCl_4_-induced rat model, TUDCA reduced collagen deposition and α-SMA expression and improved liver injury (Bai et al., 2025). TUDCA also promoted hepatocyte proliferation and liver regeneration, and GATA3 knockdown markedly attenuated its proliferative effect in BRL-3A cells (Bai et al., 2025). These findings support a role for GATA3 in TUDCA-induced regeneration, although its direct contribution to the antifibrotic effect remains unclear. In a mouse model of secondary biliary fibrosis induced by common bile duct ligation, both preventive and therapeutic administration of TUDCA reduced fibrosis and suppressed CHOP expression and pro-apoptotic signaling (Paridaens et al., 2017). These findings suggest that the protective effects of TUDCA during fibrotic liver injury may involve both the preservation of regenerative capacity and the attenuation of ERS-associated apoptosis.

### 4.4 Hepatocellular carcinoma

Hepatocellular carcinoma (HCC) is the predominant type of primary liver cancer and remains a major cause of cancer-related mortality worldwide (Bueloni et al., 2025). Experimental studies indicate that TUDCA may affect hepatocarcinogenesis through ERS-related regulation of Hippo–YAP signaling. In Mst1/2-deficient and DEN-induced mouse models, TUDCA reduced liver overgrowth and tumor formation by promoting YAP nuclear export and degradation and decreasing the expression of YAP target genes, including CTGF and Cyr61 (Wu et al., 2015). Dysregulation of PERK–eIF2α–YAP signaling was also observed in human HCC tissues, supporting the relevance of this pathway to HCC. However, these human data do not demonstrate a therapeutic effect of TUDCA (Wu et al., 2015).

TUDCA has also been implicated in the interaction between the gut microbiota and immune checkpoint therapy. In HCC-bearing mice, *Akkermansia muciniphila* enhanced the antitumor response to anti-PD-1 treatment, accompanied by increased intratumoral CD8^+^ T cells, reduced PD-L1 expression, and altered bile acid metabolism, including increased circulating TUDCA (Lan et al., 2024). Although an FXR–SHP–PD-L1 pathway was proposed to link TUDCA to these immune effects, its direct involvement was not established in that study (Lan et al., 2024). This finding suggests that bile acid remodeling may contribute to the interaction between the gut microbiota and antitumor immunity in HCC, with TUDCA emerging as a potential metabolic mediator of this process.

### 4.5 Liver ischemia-reperfusion injury

The liver IRI is a pathological phenomenon in which tissue damage worsens upon restoration of blood supply following transient ischemia. This has become one of the primary causes of hepatic dysfunction and even liver failure in clinical practice (Zeng et al., 2025). ERS is closely involved in this process, contributing to the inflammatory response and hepatocyte injury following reperfusion.

As an endogenous chemical chaperone, TUDCA has shown hepatoprotective effects in experimental ischemic liver injury. In a hemorrhagic shock/reperfusion model, TUDCA reduced serum ALT and AST levels and hepatic necrosis. TUDCA also altered the spatial distribution of ERS signaling, with increased pPERK expression in hepatocytes bordering necrotic areas, suggesting that modulation of the adaptive PERK response may contribute to its protective effect (Obert et al., 2019).

Kupffer cell activation is another important component of hepatic IRI (Gao et al., 2016). In a mouse model of warm hepatic IRI, TUDCA reduced liver injury and hepatocyte apoptosis and suppressed the release of pro-inflammatory cytokines from Kupffer cells by downregulating the IRE1α/TRAF2/NF-κB pathway (Xu et al., 2018b). In addition, bile acid signaling may participate in the response to ischemic stress. In rats subjected to hemorrhagic shock and resuscitation, endogenous hepatic TUDCA levels decreased together with SIRT1 and FXR expression. Exogenous TUDCA restored SIRT1–FXR signaling, reduced NF-κB and p53 expression and acetylation, and increased FoxM1 expression, thereby attenuating inflammation and apoptosis while promoting hepatocyte proliferation (Sun et al., 2020).

Collectively, these findings indicate that TUDCA may alleviate ischemic liver injury by coordinating cellular stress adaptation, inflammatory regulation, and hepatocyte survival and recovery through PERK-, IRE1α/TRAF2/NF-κB-, and SIRT1–FXR-related signaling.

### 4.6 Gallbladder stones

Gallbladder stones are a common biliary tract disorder closely associated with bile composition imbalances, impaired biliary drainage, and abnormal gallbladder motility (Lu et al., 2026). Currently, approximately 10–20 percent of adults worldwide are affected by this condition (Lammert et al., 2016). Although it is primarily classified as a biliary tract disorder, the formation of cholesterol stones is closely linked to liver cholesterol secretion and bile acid metabolism.

In recent years, the regulatory role of the gut-liver axis in the pathogenesis and progression of hepatobiliary diseases has garnered significant attention, with gut microbiota playing a pivotal role in bile acid regulation and metabolism. Studies indicate that mice with gallbladder stone models exhibit marked gut microbiota dysbiosis (Chen et al., 2021). Other studies have found that TUDCA intervention can alter the relative abundances of *Firmicutes* and *Bacteroidetes*, suggesting that changes in the gut microbiota may be associated with TUDCA’s regulation of bile acid and cholesterol metabolism (Lu et al., 2021).

In a murine cholesterol gallstone model, TUDCA markedly reduced gallstone formation, along with serum and hepatic cholesterol accumulation (Lu et al., 2021). On the one hand, ABCG5 and ABCG8 are key transporters responsible for cholesterol secretion from hepatic bile ducts into bile; TUDCA significantly downregulates their expression, thereby reducing cholesterol secretion into bile. On the other hand, TUDCA accelerates the conversion of cholesterol into bile acids by increasing the expression of Cyp27, a key enzyme in bile acid synthesis. This ultimately lowers the cholesterol saturation index in gallbladder bile, thereby inhibiting cholesterol crystallization and stone formation (Lu et al., 2021). Clinical observations also provide preliminary evidence of TUDCA’s effects on cholesterol and bile acid metabolism. In patients with cholecystolithiasis, short-term oral TUDCA treatment reduced serum total cholesterol, triglyceride, and LDL-C levels and was accompanied by changes in bile composition (Lu et al., 2022).

## 5 Research limitations

### 5.1 Unresolved interactions between TUDCA and the gut microbiota–bile acid axis

The gut microbiota–bile acid axis is closely involved in the enterohepatic metabolism of TUDCA, although its contribution to TUDCA activity remains incompletely understood. In an aging-related model of hepatic lipid dysmetabolism, *Campylobacter jejuni*-mediated deconjugation of TUDCA was linked to changes in FXR signaling and downstream metabolic responses (Wei et al., 2022), indicating that microbial metabolism can influence the biological activity of TUDCA. TUDCA, in turn, has been shown to alter gut microbial composition in metabolic and hepatobiliary disease models (Wang et al., 2024; Lu et al., 2021; Francini et al., 2025). The contribution of individual bacterial taxa to TUDCA biotransformation, however, has not been fully characterized, and it remains unclear whether disease-associated dysbiosis substantially alters its intestinal handling or therapeutic response. Interpretation is further complicated by the fact that microbial changes following TUDCA treatment often occur alongside changes in bile acid composition, intestinal barrier function, and host metabolism (Wang et al., 2018; Wang et al., 2024; Lu et al., 2021). The extent to which the gut microbiota directly mediates the hepatoprotective effects of TUDCA, therefore, remains to be clarified.

### 5.2 Heterogeneity of experimental models and TUDCA exposure

The experimental evidence reviewed here was generated from a broad range of models that differ in species, disease induction, severity, and treatment duration. These differences are accompanied by substantial variation in TUDCA exposure. Even among HFD-based metabolic models, oral doses range from 2.5 mg/kg/day (Wang et al., 2024) to 1,000 mg/kg/day (Wang et al., 2018). At the same time, other studies have used different treatment schedules and routes of administration, including intraperitoneal injection in hepatic ischemia–reperfusion injury (Xu et al., 2018b). Similar variation is seen *in vitro*, where TUDCA concentrations range from tens to hundreds of micromolar in hepatocyte models (Latif et al., 2022; Schoemaker et al., 2004) to the millimolar range in hypoxic HepG2 cells (Sun et al., 2020). Although these experimental conditions are useful for identifying potential mechanisms, their heterogeneity makes direct comparison across studies difficult. It limits the extent to which effective experimental concentrations can be extrapolated to clinical exposure.

This uncertainty is reinforced by the limited human pharmacokinetic data available for TUDCA. Existing studies have mainly examined PBC or biliary disposition and have provided important information on intestinal absorption, enterohepatic recycling, and the deconjugation and reconjugation of orally administered TUDCA (Setchell et al., 1996; Invernizzi et al., 1999; Rudolph et al., 2002). Much less is known about its pharmacokinetic behavior in other liver diseases or at different stages of hepatic dysfunction. As a result, the relationship among the experimentally used doses, the exposure achieved in patients, and the biological response to TUDCA remains insufficiently defined.

### 5.3 Limited clinical and comparative evidence

Clinical evidence for TUDCA remains limited in most liver diseases. Direct head-to-head comparisons with UDCA remain sparse and have been conducted mainly in PBC, with additional evidence from a small trial in patients with liver cirrhosis. Evidence in other indications is largely derived from preclinical studies or small clinical studies using short-term biochemical, imaging, or other surrogate endpoints. Some studies in fatty liver disease have also evaluated TUDCA as part of combination regimens, making its independent contribution difficult to determine. Further clinical studies should therefore move beyond short-term biochemical responses and determine whether the experimental benefits of TUDCA translate into sustained, clinically meaningful outcomes, as well as how its efficacy compares with UDCA or other established therapies in specific liver diseases. Key human studies relevant to the liver and hepatobiliary conditions discussed in this review are summarized in Table 1.

**TABLE 1:** Human studies evaluating TUDCA in liver and hepatobiliary diseases.

Study	Disease/condition	Study design	Sample size	Treatment regimen	Main findings
(Crosignani et al., 1996)	PBC	Randomized dose-response study	24	TUDCA 500, 1,000, or 1,500 mg/day for 6 months	Liver enzymes decreased with all doses, with no significant difference among dose groups; greater sustained reductions were observed with 1,000 and 1,500 mg/day
(Larghi et al., 1997)	PBC	Randomized crossover pilot study	23 enrolled; 20 analyzed	TUDCA 500 mg/day vs. UDCA 500 mg/day; 6 months each, separated by a 3-month washout	Both treatments improved cholestatic and cytolytic liver enzymes, with no significant difference between TUDCA and UDCA
(Ma et al., 2016)	PBC	Multicenter, randomized, double-blind phase III trial	199	TUDCA 250 mg three times daily vs. UDCA 250 mg three times daily for 24 weeks	ALP reduction >25% occurred in 75.97% and 80.88% of the TUDCA and UDCA groups, respectively; biochemical responses and overall safety were comparable
(Pan et al., 2013)	Liver cirrhosis	Randomized, double-blind parallel-group trial	23 enrolled; 18 analyzed	TUDCA 750 mg/day vs. UDCA 750 mg/day for 6 months	Liver enzymes improved in the TUDCA group, but serum fibrosis markers were not significantly changed in either group
(Cao et al., 2013)	NAFLD	Randomized three-arm comparative study	180	TUDCA + PPC vs. UDCA + PPC vs. PPC alone for 2 months	TUDCA- and UDCA-containing regimens improved biochemical and metabolic indices compared with PPC alone, with similar overall efficacy between TUDCA and UDCA
(Lu et al., 2022)	NAFLD	Two-group comparative clinical study	186	TUDCA 0.25 g three times daily + PPC vs. glutathione + PPC for 24 weeks	The TUDCA-containing regimen produced greater reductions in liver enzymes and CAP, with lower TC and TG at 24 weeks
(Gao et al., 2022)	Cholecystolithiasis	Randomized four-group study	96	TUDCA 5–10 mg/kg/day, probiotics, combination therapy, or no drug intervention for 2 weeks	TUDCA-containing treatment altered serum biochemical indices and bile composition; combination therapy did not provide additional benefit over the individual interventions

Abbreviations: ALP, alkaline phosphatase; CAP, controlled attenuation parameter; NAFLD, non-alcoholic fatty liver disease; PBC, primary biliary cholangitis; PPC, polyene phosphatidylcholine; TC, total cholesterol; TG, triglycerides; TUDCA, tauroursodeoxycholic acid; UDCA, ursodeoxycholic acid.

## 6 Discussion

Although TUDCA has been implicated in multiple pathways involved in liver injury, much of the available evidence converges on its ability to alleviate cellular stress and preserve hepatocyte survival. Modulation of ERS and the unfolded protein response is frequently accompanied by changes in inflammatory signaling, oxidative stress, and apoptosis (Latif et al., 2022; Schoemaker et al., 2004; Song et al., 2023), suggesting that these effects are closely interconnected rather than independent actions of TUDCA. This may also account for the protective effects observed across different experimental models.

As research has expanded, TUDCA has also been linked to bile acid signaling, lipid metabolism, liver regeneration, and the gut–liver axis. These findings have broadened the understanding of its biological effects, although some are more context-dependent. FXR-related responses, for example, differ between metabolic and cholestatic models (Wang et al., 2024; Song et al., 2023). Changes in gut microbiota and bile acid composition have also been observed after TUDCA treatment (Wang et al., 2018; Wang et al., 2024; Lu et al., 2021). However, their contribution to its hepatoprotective effects has not been clearly distinguished from accompanying metabolic changes.

This expanding mechanistic evidence has not yet been matched by clinical studies. The most substantial clinical data are currently available for PBC, where TUDCA has shown short-term biochemical efficacy and safety comparable to UDCA (Ma et al., 2016). For other liver diseases, evidence remains largely experimental or is based on limited clinical observations. The proposed role of TUDCA in HCC is particularly preliminary. Its effects on Hippo/YAP signaling have been demonstrated in experimental models (Wu et al., 2015), whereas its possible involvement in microbiota-associated responses to anti-PD-1 therapy remains to be established (Lan et al., 2024). Further studies are needed to clarify which of these experimental findings are clinically relevant and whether they translate into sustained therapeutic benefit.
